# Acknowledgement to Reviewers of *Life* in 2013

**DOI:** 10.3390/life4010105

**Published:** 2014-02-27

**Authors:** Life Editorial Office

**Affiliations:** MDPI AG, Klybeckstrasse 64, CH-4057 Basel, Switzerland

The editors of *Life* would like to express their sincere gratitude to the following reviewers for assessing manuscripts in 2013:
Adamatzky, AndrewHolm, Nils G.Pletser, VladimirBarbieri, RobertoHubera, HaraldRachel, ReinhardBenner, S. A.Panagiotopoulos, I. A.Rengel, M.Bowden, StephenIslam, SaidulRussell, Michael J.Bremer, ErhardItoh, TakashiSandoval, GeorginaBrown, Grant E.Jebaraj, Cathrine SumathiSanz, José LuisBurton, Aaron S.Klatt, Christian G.Savka, Michael A.Carter-Bond, Jade C.Kochetkova, Tatiana V.Schild, Rudolf E.Cataldo, FrancoKohls, KatharinaSchloemann, MichaelChalmers, J.Kolb, VeraSchüler, DirkCintas, PedroKönnyű, BalázsSchwartz, AlanClark, Torin K.Lauga, BeatriceSelbmann, LauraCorcelli, AngelaLi, Yi-LiangSephton, MarkDa Luz, M. G. E.Lins, UlyssisServín-Garcidueñas, Luis E.Danchin, AntoineLondon, Robert E.Sheldon, Robert B.Di Mauro, ErnestoLovejoy, ConnieSimpson, H. N.Durston, KirkLowenstein, Tim K.Smith, Mark A.Falteisek, LukášMa, Edmond Dik-LungSodupe, MarionaForgan, DuncanMaeng, Pil JaeTian, GeFoster, Jamie S.Mann, StephenUgliengo, PieroFrankel, RichardMartel, JanVargas, CarmenGentili, Pier LuigiMcIntosh, Professor Andyvon Bloh, WernerGonzalez, JuanMills, Kenneth V.Wang, WeiGoswami, NanduNewstead, SimonWilliams, TimothyHaghighipour, NaderPagaling, EulynWylezich, ClaudiaHässig, M.Pansé, VikramZhou, PeiHeeb, StephanPascal, RobertHiggs, Pg.Pilat-Lohinger, Elke


